# A study on the symptomatic association between depressive symptoms and rumination in college students–based on latent profile analysis and network analysis

**DOI:** 10.3389/fpsyg.2026.1875520

**Published:** 2026-08-12

**Authors:** Shuai Liu, Bingxin Cai

**Affiliations:** 1 School of Educational Science, Xinyang Normal University, Xinyang, China; 2 Mental Health Center, Xinyang Normal University, Xinyang, China

**Keywords:** college students, depressive symptoms, latent profile analysis, network analysis, rumination

## Abstract

**Background:**

In recent years, depressive symptoms are common among college students worldwide and constitute an important public health concern, with their associated disease burden drawing increasing attention from researchers and public health practitioners. Although rumination has been widely recognized as a key cognitive vulnerability factor for depressive symptoms, it is often conceptualized as a unitary construct, thereby neglecting the functional distinctions and potential heterogeneity across different emotional contents (e.g., sadness and anger) embedded within the ruminative process. Accordingly, a re-examination of the association linking emotion-specific rumination to depressive symptoms at the symptom level, from an emotion-specific perspective, is warranted.

**Methods:**

A total of 8,729 college students completed the Anger and Sadness Rumination Questionnaire and the Patient Health Questionnaire-9 (PHQ-9). Latent profile analysis (LPA) was conducted using item-level indicators to identify heterogeneous response profiles. A regularized partial-correlation network was then estimated within the elevated-depressive-symptom profile using 22 rumination items and one aggregate PHQ-9 total-score node. Gender-group networks were compared using the Network Comparison Test.

**Results:**

The LPA identified three distinct and heterogeneous profiles: an elevated-depressive-symptom profile characterized by high anger rumination and low sadness rumination, a low-depressive-symptom profile characterized by low anger rumination and high sadness rumination, and a moderate-depressive-symptom profile with moderate levels of rumination. Within the elevated-depressive-symptom profile, several anger- and sadness-rumination items showed relatively high observed expected-influence estimates in a network comprising 22 rumination items and one aggregate PHQ-9 total-score node. These findings represent conditional associations between rumination items and overall depressive-symptom severity rather than a network among individual depressive symptoms. Exploratory gender-stratified analyses suggested descriptive differences in node centrality patterns.

**Conclusion:**

The findings underscore substantial heterogeneity in depressive symptom and rumination patterns among college students and highlight the relative prominence of several emotion-specific rumination items within the estimated network. These findings emphasize the value of distinguishing emotion-specific rumination processes and integrating latent profile and network approaches to better characterize the complexity of depressive symptom patterns among college students.

## 1 Introduction

Depressive symptoms are common among college students and constitute an important global public health concern (Pedrelli et al., 2015; Eisenberg et al., 2007). College students are particularly vulnerable to elevated depressive symptoms because they face concurrent developmental challenges, including academic pressure, interpersonal adjustment, and identity transitions. Such symptoms are associated with emotional distress, impaired academic functioning, social withdrawal, and poorer physical health, including sleep problems and cardiovascular risk (Tonhajzerova et al., 2022). Importantly, the present study concerns depressive symptoms assessed by a self-report questionnaire in a non-clinical college sample; it does not assess or make inferences about clinically diagnosed depressive disorders.

Recent research indicates that depressive symptoms among college students are complex and multidimensional in nature. Cross-sectional studies have consistently reported a high prevalence of depressive symptoms in this population, with systematic variations observed across factors such as gender, socioeconomic status, and academic stress. Overall, prevalence rates demonstrate a pattern of either continuous increase or fluctuation over time (Jenaro et al., 2021). Complementing these findings, longitudinal studies suggest depressive symptoms are not static but rather evolve dynamically through interactions with related variables, including anxiety, life satisfaction, and environmental adaptation. These studies further highlight substantial interindividual variability in developmental trajectories (Tsitsas et al., 2019; Han et al., 2025). At the symptom level, depressive symptoms in college students reflect the interplay of multiple dimensions, including low mood, anhedonia, and cognitive difficulties. These core symptoms are often accompanied by related features such as interpersonal stress, loneliness, and changes in behavioral patterns, which co-occur in diverse configurations across individuals (Chen et al., 2026). Moreover, developmental and process-oriented research suggests that depressive symptoms during adolescence and emerging adulthood exhibit both continuity and fluctuation. Individuals may follow distinct developmental trajectories, including persistent high symptom levels, remission, or recurrent episodes (Whittle et al., 2014; Brière et al., 2013). Therefore, identifying cognitive processes that contribute to individual differences in depressive symptom patterns may provide important insights into the heterogeneity of depressive symptoms among college students.

Among various cognitive vulnerability factors, rumination is a prominent cognitive processing mechanism that is highly prevalent among college students and exhibits relatively stable yet complex psychological characteristics (Zawadzki et al., 2013). Cross-sectional research has demonstrated that rumination is significantly associated with a range of mental health indicators, including depressive symptoms, anxiety, and sleep quality. Moreover, it has been shown to mediate the relationships between contextual and intrapersonal factors—such as social support and emotion regulation capacity—and psychological adaptation, underscoring its critical bridging role (Hu et al., 2025; Ehring and Ehlers, 2014). Phenomenologically, rumination is characterized by repetitive, negative, and self-focused thinking, whereby individuals persistently dwell on adverse emotional experiences and their underlying causes, rather than engaging in problem-solving or adaptive regulatory strategies (Spasojević and Alloy, 2001).

A substantial body of research indicates that rumination not only predicts the onset of depressive symptoms but also contributes to their maintenance and exacerbation over time (Hankin, 2008). Evidence from systematic reviews and meta-analyses further supports a stable, moderate positive association between rumination and depressive symptoms, with this relationship being moderated by metacognitive factors (Cano-Lopez et al., 2022). The relationship between rumination and depressive symptoms may involve multiple interrelated cognitive, emotional, and interpersonal processes. At the cognitive level, rumination amplifies negative information processing biases, leading individuals to repeatedly focus on adverse memories and experiences of failure, thereby reinforcing negative self-evaluations and feelings of helplessness. In this context, rumination mediates the association between cognitive biases—such as negative attributional style and memory bias—and depressive symptoms, whereby greater engagement in negative repetitive thinking increases the likelihood that these biases will translate into depressive experiences (Wisco et al., 2014). At the emotional level, rumination is associated with prolonged negative affect and difficulties in emotional recovery, which may contribute to more persistent depressive symptoms. At the interpersonal level, longitudinal evidence suggests that rumination not only directly predicts depressive symptoms but also indirectly exacerbates them by reducing perceived social support and increasing interpersonal stress (Yin et al., 2025).

Furthermore, accumulating evidence suggests that rumination is not a unitary cognitive process but rather consists of distinct forms characterized by different emotional contents and regulatory functions. Among these forms, sadness rumination and anger rumination have received particular attention because they represent two theoretically distinguishable forms of emotion-specific rumination associated with different emotional experiences. Although depressive symptoms, sadness rumination, and anger rumination share certain cognitive characteristics as forms of repetitive negative thinking, sadness rumination and anger rumination differ in their emotional focus and are therefore considered related but distinct constructs. Sadness rumination refers to repetitive attention toward experiences of loss, personal shortcomings, and internal emotional distress. This inward-focused cognitive process may strengthen negative self-referential thinking, reduce behavioral activation, and reinforce feelings of helplessness, thereby closely linking to depressive symptom experiences (Conway et al., 2000; Lyubomirsky and Tkach, 2004). In contrast, anger rumination involves repetitive thinking about anger-provoking events, perceived injustice, and interpersonal offenses. Unlike sadness rumination, which primarily reflects internalized negative self-focus, anger rumination is characterized by heightened emotional arousal and externally oriented cognitive processing, which may increase hostility, interpersonal conflict, and difficulties in emotion regulation (Peled and Moretti, 2007, 2010). Therefore, examining sadness rumination and anger rumination simultaneously provides an opportunity to characterize different affective-cognitive patterns associated with depressive symptoms. Sadness rumination may represent an internal cognitive-affective pattern characterized by negative self-evaluation and emotional persistence, whereas anger rumination may reflect a more externally oriented cognitive-affective pattern characterized by sustained emotional arousal and interpersonal stress. Investigating these two emotion-specific forms of rumination may thus provide a more comprehensive understanding of heterogeneity in depressive symptom patterns among college students.

Accordingly, conceptualizing emotion-related rumination as a homogeneous construct may obscure the distinct roles that different emotional contents may play in depressive symptom patterns. Emerging evidence suggests that different forms of content-specific rumination may show distinct associations with depressive symptoms. On the one hand, sadness rumination appears to show a closer association with depressive symptoms and has been implicated in persistent negative mood states (Jahanitabesh et al., 2019). On the other hand, anger rumination may be associated with heightened emotional arousal and interpersonal conflict, which may represent pathways linking anger-related cognitive processes with depressive symptoms (Besharat et al., 2013). Therefore, emotion-specific rumination may provide a more precise framework for identifying heterogeneous depressive symptom patterns and understanding symptom-level mechanisms. Although sadness rumination and anger rumination share variance attributable to repetitive negative thinking, each captures distinct emotional-cognitive processes and therefore provides unique information beyond their shared cognitive variance.

Given the multidimensional nature and complex co-occurrence of depressive symptoms and rumination, it is likely that distinct “symptom–cognition” interaction patterns exist within the college student population. Consequently, there is a need to adopt person-centered approaches, such as latent profile analysis (LPA), to identify potentially heterogeneous subgroups. However, although LPA is effective for capturing between-person heterogeneity, it remains limited in its ability to elucidate the interactions among symptoms within identified subgroups. In contrast, the network theory of psychopathology conceptualizes depression as a complex system of interacting symptoms, emphasizing direct symptom-to-symptom associations and their potential relevance to symptom persistence (Fried and Cramer, 2017; Hevey, 2018). Within this framework, examining node centrality may provide useful information for understanding symptom organization and generating hypotheses for future intervention research. Therefore, integrating LPA with symptom network analysis offers a complementary approach that advances understanding from the identification of heterogeneity to the delineation of symptom-level structures.

Building on this foundation, it is essential to further examine gender differences. As a key factor shaping emotional processing, gender plays a critical role in the co-occurrence of rumination and depressive symptoms. According to social role theory, sociocultural norms systematically influence how individuals of different genders experience, express, and regulate emotions (Eagly and Wood, 2012; Koenig and Eagly, 2014). Women are generally encouraged to attend to and express internal emotional states, whereas men are more likely to be constrained by norms that discourage the expression of vulnerability (Kretchmar, 2011). Existing research consistently indicates that women tend to report higher levels of rumination and depressive symptoms than men. However, at the level of symptom networks, gender differences may be reflected more in structural configurations than in overall severity (Smith et al., 2008). In other words, gender may be associated with variations in the relative importance of different emotional components within symptom networks. Despite this, empirical evidence regarding how gender shapes symptom network structures within high-risk subgroups remains limited.

Given the limited theoretical and empirical evidence, there was insufficient prior evidence to justify specific directional hypotheses regarding latent profile membership or network centrality. Regarding the joint heterogeneity of depressive symptoms, sadness rumination, and anger rumination, the present study adopts an exploratory, data-driven approach rather than testing predefined directional hypotheses. The study was not preregistered. Accordingly, the analyses should be interpreted as exploratory rather than confirmatory and should be viewed as hypothesis-generating, requiring independent replication in future studies. Therefore, distinguishing sadness rumination from anger rumination may provide a more comprehensive understanding of the heterogeneous cognitive-emotional processes associated with depressive symptoms among college students. Specifically, (1) latent profile analysis is used to identify subgroups based on sadness rumination, anger rumination, and depressive symptoms; (2) a symptom network is estimated within the subgroup exhibiting elevated depressive symptoms to identify nodes with relatively high expected influence; and (3) gender-stratified networks are compared to explore potential structural differences.

## 2 Subjects and methods

### 2.1 Participants

Before participating in the survey, all participants were informed of the study purpose, their right to participate voluntarily, and the confidentiality of their responses. Electronic informed consent was obtained from all participants prior to data collection. Participation was entirely voluntary, and no financial compensation or other incentives were provided. Data were collected through the Wenjuanxing online questionnaire system using a convenience sampling strategy. The participants were undergraduate students from different academic levels at a university located in Henan Province, China. A total of 9,006 questionnaires were initially collected. After data screening, 277 invalid responses were excluded based on predefined criteria, including unusually short completion times and substantial missing data. Consequently, 8,729 valid responses were retained for the final analysis, yielding an effective response rate of 96.92%. The final sample consisted of 2,669 male participants (30.6%) and 6,060 female participants (69.4%), with a mean age of 19.59 years.

### 2.2 Measures

#### 2.2.1 Anger and sadness rumination questionnaire

Rumination was evaluated using the Anger and Sadness Rumination Questionnaire developed by Peled and Moretti (2007). This measure comprises 22 items and captures two dimensions: sadness rumination (e.g., “I frequently dwell on past events that elicited feelings of sadness”) and anger-related rumination (e.g., “I frequently dwell on past events that elicited feelings of anger”). All items are rated on a 5-point Likert-type scale ranging from 1 (never) to 5 (always). Scores are computed as the mean of items within each subscale, with higher values indicating stronger tendencies toward sadness or anger rumination. In the present study, the instrument demonstrated excellent internal reliability (Cronbach’s α = 0.98).

#### 2.2.2 Patient health Questionnaire-9 (PHQ-9)

Depressive symptomatology was assessed using the Patient Health Questionnaire-9 (PHQ-9) developed by Kroenke et al. (2001), which measures the frequency of depressive experiences over the preceding 2 weeks. The instrument contains nine items, each scored on a 4-point Likert-type scale from 0 (not at all) to 3 (nearly every day), producing a total score ranging from 0 to 27. Higher total scores indicate greater depressive symptom severity. Prior studies have confirmed that the PHQ-9 demonstrates satisfactory reliability and discriminant validity in university student populations . In the current sample, the scale exhibited strong internal consistency (Cronbach’s α = 0.91).

### 2.3 Statistical methods

#### 2.3.1 Latent profile analysis (LPA)

To identify latent subgroups of rumination and depressive symptoms among college students, latent profile analysis (LPA) was performed using Mplus 8.3. Item-level responses from the rumination and depressive symptom scales were specified as manifest indicators. Model estimation proceeded in a stepwise manner, starting with a single-class solution and gradually increasing the number of latent classes.

The optimal number of latent profiles was selected based on a combination of multiple model fit indices, including the Akaike Information Criterion (AIC), Bayesian Information Criterion (BIC), sample-size adjusted BIC (aBIC), entropy, the Lo–Mendell–Rubin adjusted likelihood ratio test (LMR-LRT), and the bootstrap likelihood ratio test (BLRT). Lower values of AIC, BIC, and aBIC indicate superior model fit, whereas entropy values approaching 1.0 reflect higher classification precision; values above 0.80 are generally interpreted as indicating adequate to high classification accuracy (Lubke and Muthén, 2007).

Statistically significant *p*-values (*p* < 0.05) for the LMR-LRT and BLRT indicate that a model with k classes fits the data significantly better than a model with k–1 classes (Nylund et al., 2007). In addition to statistical fit indices, substantive interpretability and model parsimony were also considered in determining the final latent class solution.

#### 2.3.2 Network analysis

A 23-node Gaussian graphical model was estimated using graphical LASSO regularization with Extended Bayesian Information Criterion model selection, implemented in the bootnet package in R. The EBIC tuning hyperparameter was set to (γ = 0.50). Because the network included 22 ordinal rumination items and one aggregate PHQ-9 total-score node, a Spearman rank-correlation matrix was used. The ordinal item responses were therefore incorporated according to their rank ordering without assuming equal intervals or multivariate normality. Pairwise-complete estimation was specified, although all 2,000 participants had complete data on the 23 network variables. No additional threshold was applied after EBICglasso estimation; edges were retained when their regularized partial-correlation estimates were nonzero.

Edge-weight accuracy was evaluated using 1,000 nonparametric bootstrap samples. Differences between expected-influence estimates were examined using bootstrapped 95% confidence intervals for pairwise centrality differences. The stability of expected influence was evaluated using 1,000 case-dropping bootstrap samples and quantified using the correlation-stability coefficient.

Gender networks were compared using the Network Comparison Test with 1,000 permutations. The analysis tested network-structure invariance, global-strength invariance, individual edge differences, and expected-influence differences. Holm correction was applied to local edge and expected-influence comparisons to control for multiple testing.

#### 2.3.3 Network comparison test (NCT)

To investigate potential gender-related differences in network structures, the Network Comparison Test (NCT) was implemented using the NetworkComparisonTest package. This permutation-based analytical approach allows for formal statistical comparisons of network models across independent groups. Specifically, global strength invariance (i.e., overall network connectivity) and structural invariance of the network were evaluated at the global level. In addition, group differences at the local level were examined in terms of node expected influence and individual edge weights (van Borkulo et al., 2023). Because this study was exploratory rather than hypothesis-driven, no *a priori* hypotheses regarding the number of latent profiles, the centrality of individual network nodes, or gender-specific network structures were specified before the analyses.

## 3 Results

### 3.1 Latent profile analysis of rumination and depressive symptoms among college students

Descriptive statistics of the rumination items are presented in Table 1. Within latent profile analysis (LPA), the selection of the optimal model necessitates a comprehensive appraisal of statistical fit criteria alongside substantive theoretical interpretability. As shown in Table 2, an increase in the number of latent classes was accompanied by progressively reduced values of the Akaike Information Criterion (AIC), Bayesian Information Criterion (BIC), and sample-size adjusted BIC (aBIC), indicating incremental improvements in model fit (Wang and Bi, 2018).

**TABLE 1:** Descriptive statistics and analysis of variance.

Abbreviations	Content	Means	SD	Skewness	Kurtosis
SRQ1	I always recall past experiences that made me sad.	2.121	0.923	0.309	−0.496
SRQ2	I find it hard to stop thinking about how sad I am.	1.891	0.877	0.761	0.201
SRQ3	I keep thinking about the reasons for my sadness.	2.018	0.932	0.609	−0.146
SRQ4	When I think about my sadness, I feel even worse.	2.167	0.965	0.341	−0.569
SRQ5	I can’t stop thinking about why I’m so sad and find it hard to focus on other things.	1.903	0.886	0.806	0.353
SRQ6	I search my mind for past events and experiences to explain my current feelings of sadness.	2.063	0.944	0.521	−0.301
SRQ7	If something upsets me, I keep going over it in my mind.	2.071	0.935	0.516	−0.301
SRQ8	I am tired of always thinking about myself and the reasons for my sadness.	2.022	0.996	0.834	0.287
SRQ9	Whenever I feel sad, I keep thinking about it for a long time.	2.074	0.950	0.547	−0.274
SRQ10	I recall past events that still make me sad.	2.087	0.927	0.505	−0.147
SRQ11	The more I think about my sadness, the sadder I become.	2.084	0.955	0.558	−0.227
ARQ12	I always recall past experiences that made me angry.	1.940	0.870	0.714	0.294
ARQ13	I find it hard to stop thinking about how angry I am.	1.895	0.850	0.852	0.756
ARQ14	Other people are worthless except for myself.	1.963	0.887	0.662	0.015
ARQ15	I keep thinking about the reasons for my anger.	2.111	0.918	0.633	0.199
ARQ16	I can’t stop thinking about why I’m so angry and find it hard to focus on other things.	1.903	0.816	0.654	0.129
ARQ17	I search my mind for past events and experiences to explain my current feelings of anger.	2.103	0.903	0.400	−0.392
ARQ18	If something makes me angry, I keep going over it in my mind.	2.035	0.885	0.543	−0.098
ARQ19	I am tired of always thinking about myself and the reasons for my anger.	2.034	0.942	0.806	0.475
ARQ20	Whenever I feel angry, I keep thinking about it for a long time.	1.998	0.875	0.569	−0.044
ARQ21	I often recall past events that still make me angry.	1.952	0.893	0.629	0.179
ARQ22	The more I think about my anger, the angrier I become.	2.012	0.892	0.612	−0.025
PHQ	PHQ total score	11.099	3.127	2.710	11.462

**TABLE 2:** Results of the latent profile analysis of depressive symptoms and rumination.

c						*p*-value		
Model	[Log(L)]	AIC	BIC	aBIC	Entropy	LMRT	BLRT	Class proportions
1	−369291.965	738707.930	739146.544	738949.519	–			
2	−339719.213	679626.425	680291.419	679992.704	0.919	*p* < 0.001	*p* < 0.001	0.532/0.468
3	**−328692.692**	**657637.384**	**658528.759**	**658128.353**	**0.924**	***p* < 0.001**	***p* < 0.001**	**0.269/0.224/0.507**
4	−324263.710	648843.420	649961.176	649459.080	0.908	*p* < 0.001	*p* < 0.001	0.330/0.147/0.409/0.114

AIC, Akaike’s Information Criterion; BIC, Bayesian Information Criterion; aBIC, adjusted Bayesian Information Criterion; Entropy, Information Entropy; LMR, Lo-Mendell-Rubin Likelihood Ratio Test; BLRT, bootstrap likelihood ratio test. The bold values represent the optimal model based on model fit indices.

Models specifying two to four classes produced entropy values exceeding 0.80, indicating adequate classification quality. Furthermore, the Lo–Mendell–Rubin adjusted likelihood ratio test (LMR-LRT) and bootstrap likelihood ratio test (BLRT) were statistically significant for these solutions.

**FIGURE 1:**
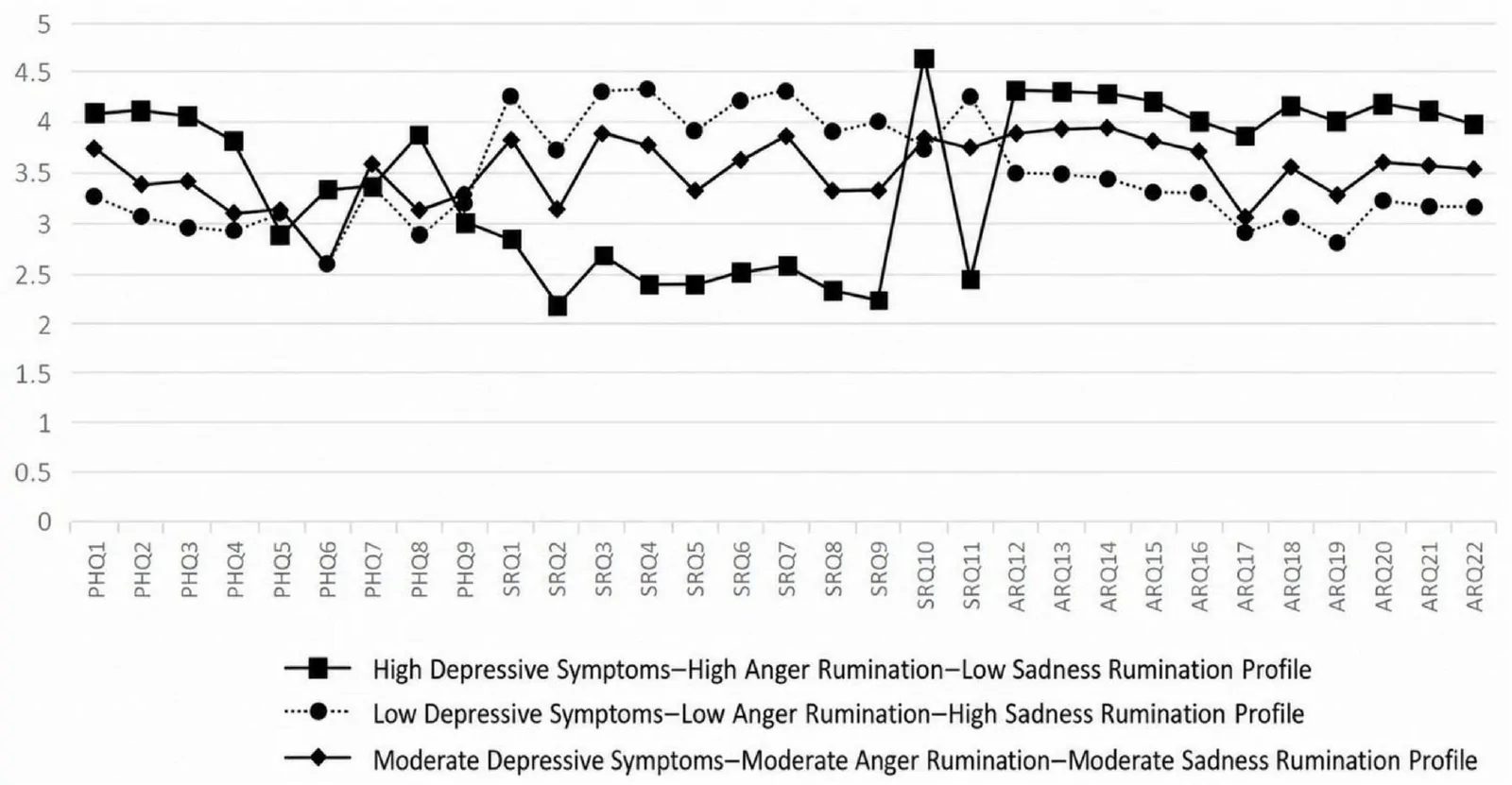
Estimated item means across the three latent profiles.

Considering model parsimony, classification quality, and substantive interpretability of latent class solutions, the three-class model was retained as the optimal representation. The mean values of each latent class on depressive symptom and rumination indicators are displayed in Figure 1.

Class 1 (*n* = 2,348) was characterized by elevated levels of depressive symptoms and anger rumination, coupled with relatively lower levels of sadness rumination, and was therefore labeled the high depressive symptoms–high anger rumination–low sadness rumination profile. Class 2 (*n* = 1,955) showed lower levels of both depressive symptoms and anger rumination but higher levels of sadness rumination, and was defined as the low depressive symptoms–low anger rumination–high sadness rumination profile. Class 3 (*n* = 4,426) exhibited intermediate levels across depressive symptoms, anger rumination, and sadness rumination, and was accordingly classified as the moderate depressive symptoms–moderate anger rumination–moderate sadness rumination profile.

### 3.2 Network analysis within the elevated depressive symptoms–high anger rumination–low sadness rumination profile

The 23-node network had 253 possible undirected edges. Following EBICglasso regularization, 142 edges were retained with nonzero weights and 111 edges were estimated as zero, corresponding to a network density of 56.1%. Of the 142 retained edges, 128 were positive and 14 were negative, with weights ranging from −0.040 to 0.343. The strongest positive associations were observed between ARQ12 and ARQ13 (*w* = 0.343), ARQ20 and ARQ21 (*w* = 0.299), ARQ18 and ARQ20 (*w* = 0.284), SRQ1 and SRQ2 (*w* = 0.276), and ARQ21 and ARQ22 (*w* = 0.275). The complete regularized edge-weight estimates are reported in Supplementary Tables 1–3.

**FIGURE 2:**
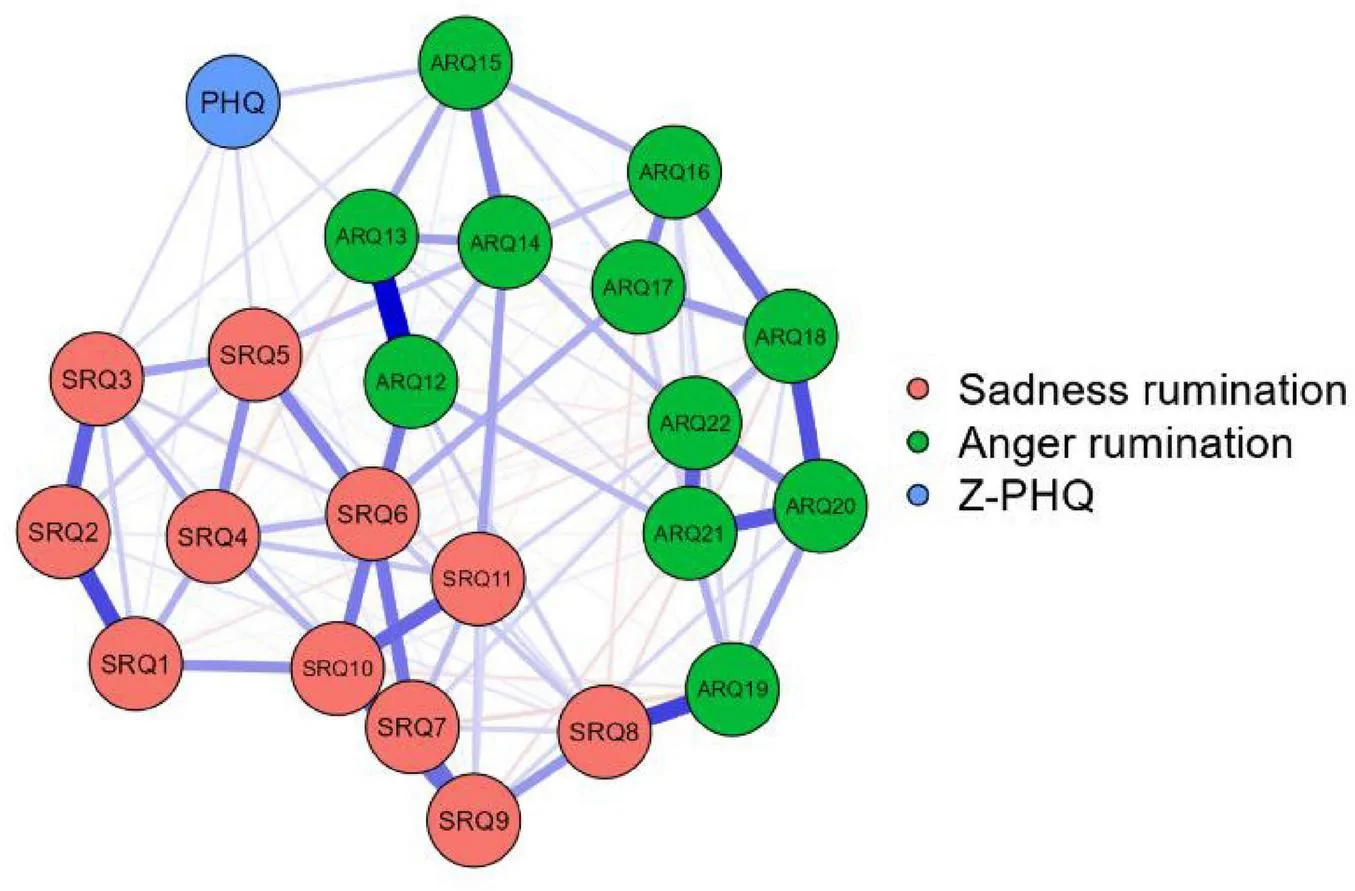
Network structures of the high depression–high anger–low sadness group.

To identify central rumination items associated with overall depressive-symptom severity within the high-risk subgroup (i.e., high depressive symptoms–high anger rumination–low sadness rumination), a network analysis was performed using individual rumination items and aggregate depressive-symptom severity. The estimated network topology is displayed in Figure 2.

**FIGURE 3:**
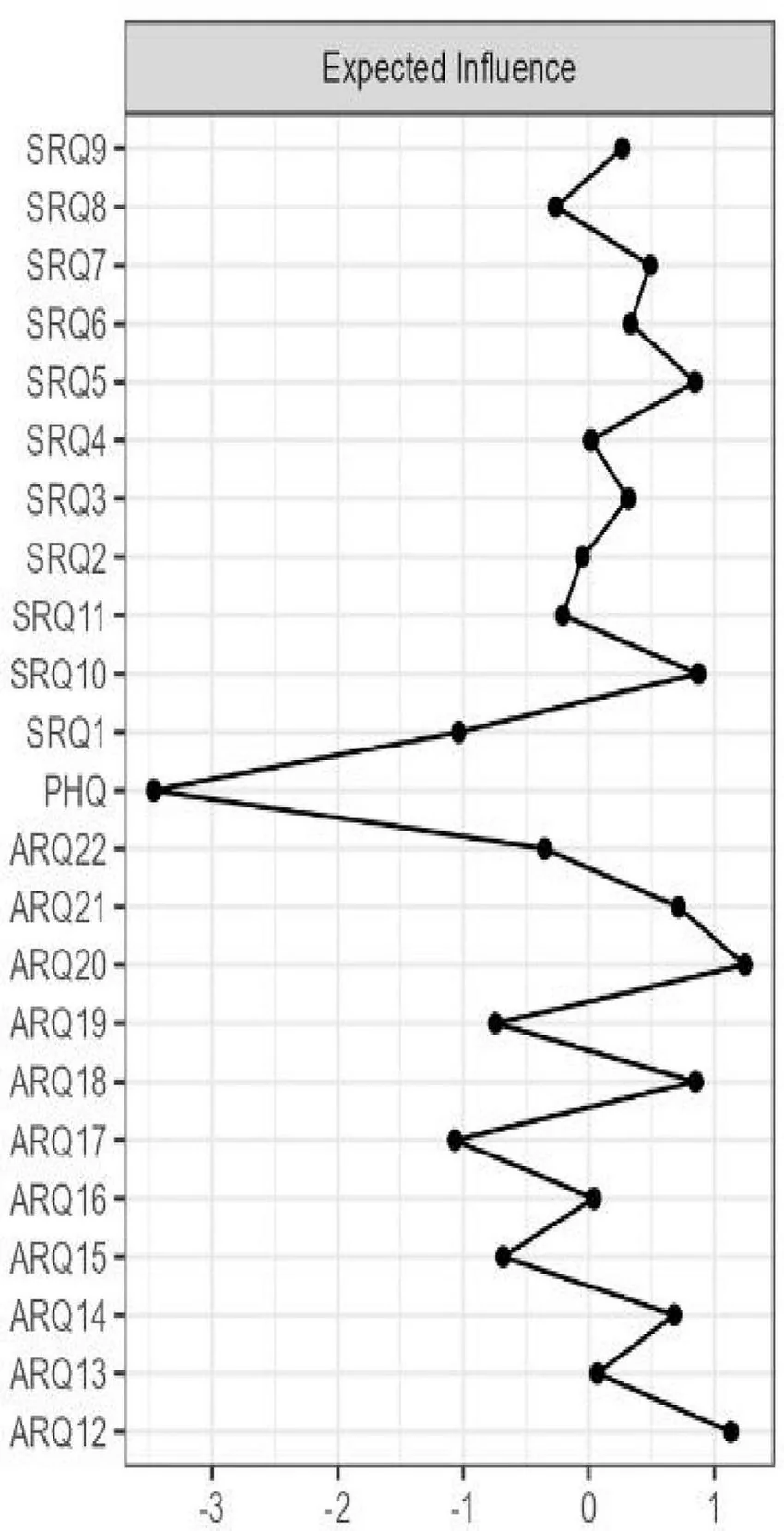
Expected influence of the high depression–high anger–low sadness group.

Expected influence identified ARQ20 (“Whenever I feel angry, I continue to think about it for a period of time”), ARQ12 (“I often recall past experiences that made me feel angry”), and SRQ10 (“I recall past events that still make me feel sad”) as having the highest expected-influence estimates in the network (Figure 3). However, bootstrap centrality-difference tests indicated that these expected-influence estimates did not differ significantly from one another. Therefore, these nodes should be interpreted as having the highest observed expected-influence estimates in the present sample rather than being definitively more central than other nodes. Their relatively high expected-influence values indicate that these items may warrant further investigation in longitudinal and experimental research.

Finally, the accuracy of edge weight estimates and the stability of centrality metrics were examined. The nonparametric bootstrap results showed that the strongest edge estimates had 95% confidence intervals that remained above zero, although confidence intervals overlapped for several edges, indicating that fine-grained differences in edge rankings should be interpreted cautiously. The case-dropping bootstrap yielded a correlation-stability coefficient of 0.75 for expected influence, indicating good stability. The edge-weight confidence intervals, case-dropping stability curve, and bootstrapped centrality-difference tests are presented in Supplementary Figures 1–3.

### 3.3 Comparison of network analysis between male and female participants in the high depressive symptoms–high anger–low sadness rumination subgroup

Based on the results of the latent profile analysis, gender-group networks were estimated within the high depressive symptoms–high anger rumination–low sadness rumination profile, which included 1,384 female and 616 male participants. Following EBICglasso regularization, the female network retained 146 of the 253 possible edges, whereas the male network retained 127 edges, corresponding to network densities of 57.7% and 50.2%, respectively.

**FIGURE 4:**
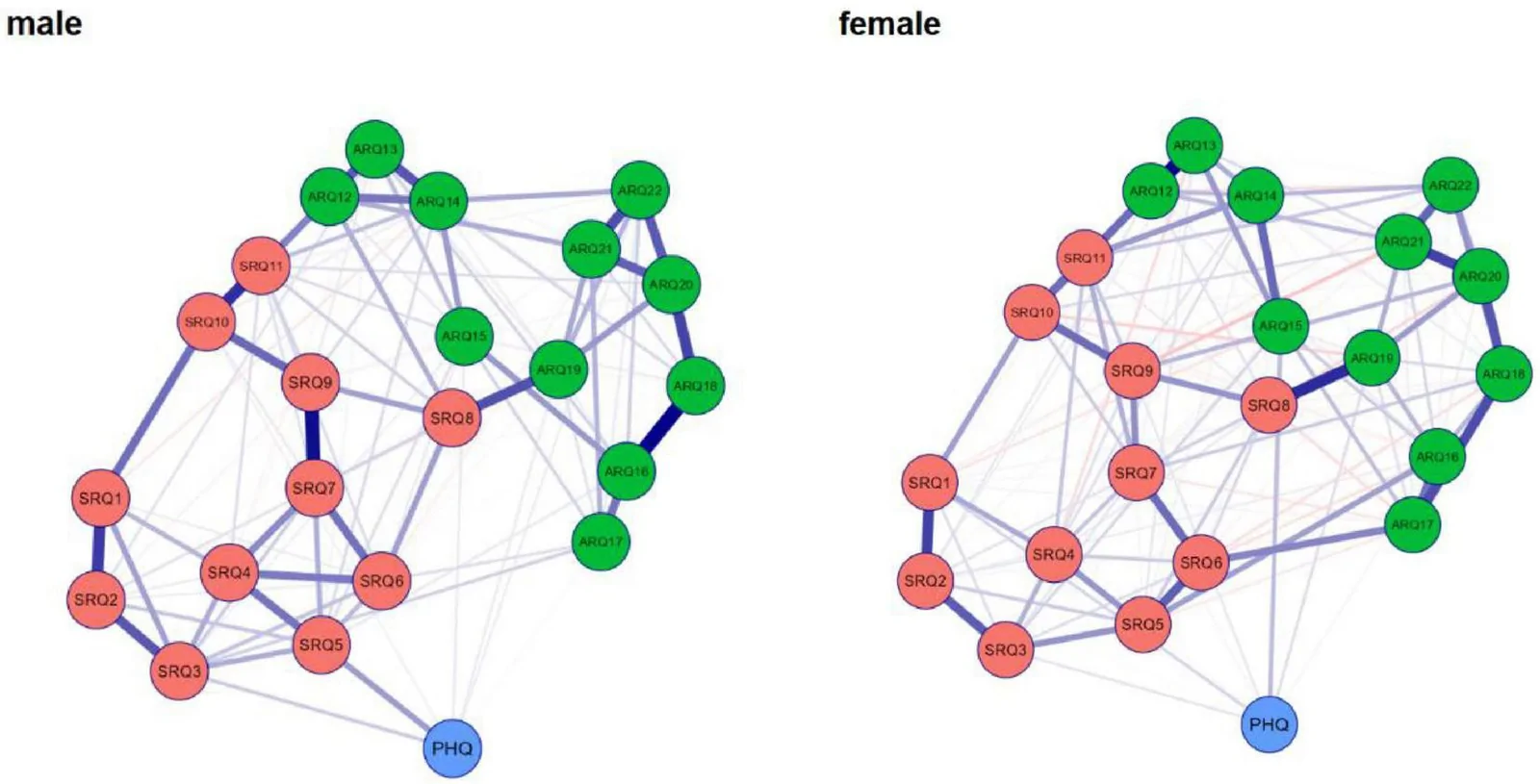
Network structures of male and female networks.

**FIGURE 5:**
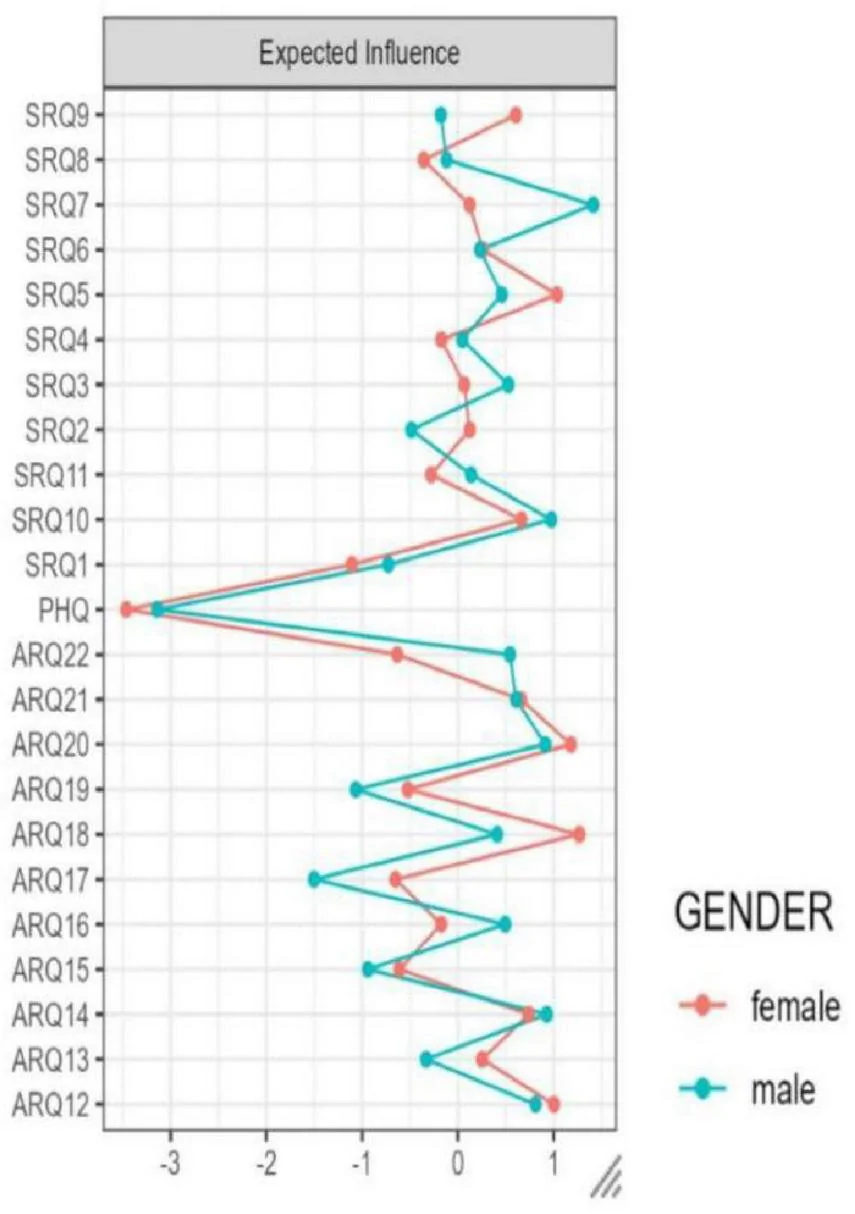
Expected influence of female and male networks.

Descriptive differences in node centrality patterns were observed between the male and female networks. As illustrated in Figures 4, 5, in the male network, the nodes with relatively higher observed expected-influence estimates were SRQ7 (“If something happens that makes me feel sad, I repeatedly dwell on it in my mind”), SRQ10 (“I recall certain past events that still make me feel sad”), and ARQ14 (“Everyone else is worthless except me”). In contrast, the female network showed relatively high observed expected-influence estimates for ARQ18 (“If something happens that makes me feel angry, I dwell on it in my mind”), ARQ20 (“Whenever I feel angry, I keep thinking about it for a period of time”), and SRQ5 (“I keep wondering why I feel so sad, and it is difficult for me to think about other things”). These differences should be interpreted as descriptive variations in node centrality patterns rather than evidence of distinct gender-specific causal pathways.

**FIGURE 6:**
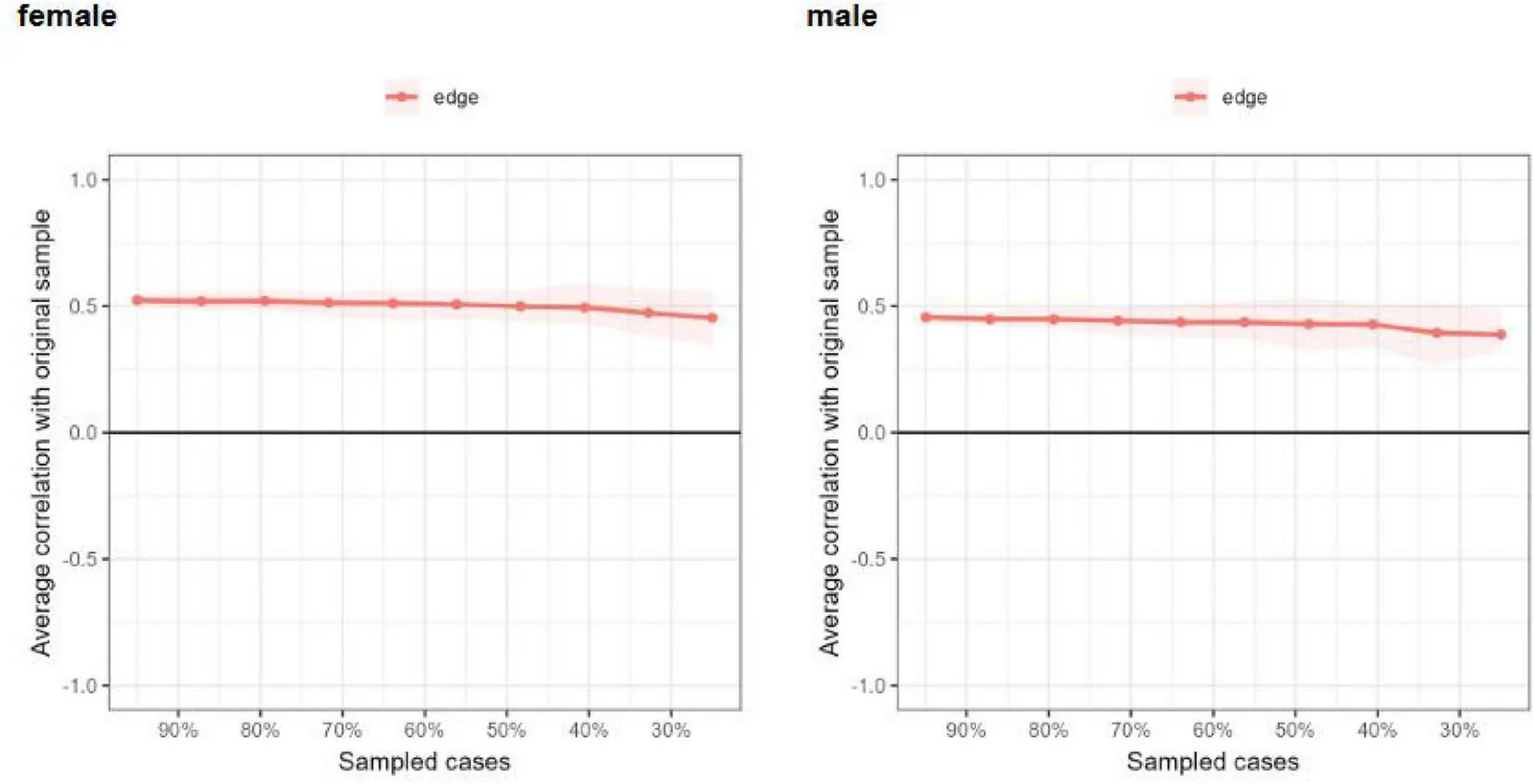
Stability of the gender-stratified network models.

Overall, although both networks showed similar edge counts, specific centrality patterns differed between gender groups. Stability analyses indicated acceptable centrality stability in both networks (CS_male = 0.438; CS_female = 0.516), exceeding the recommended minimum of 0.25 (Epskamp et al., 2018; Isvoranu et al., 2022). Because the female subgroup was substantially larger than the male subgroup, the female estimates may nevertheless be more precise; therefore, these descriptive differences should be interpreted as exploratory findings pending replication.

## 4 Discussion

Based on a large non-clinical sample of college students and integrating latent profile analysis with symptom network analysis, this study examined associations between emotion-specific rumination and self-reported depressive symptoms, as well as exploratory gender-related differences, from the complementary perspectives of individual heterogeneity and symptom structure. The findings concern symptom variation in the general college population and should not be generalized to clinically diagnosed depression.

First, rumination and depressive symptoms exhibited substantial individual variability among college students and could be classified into three distinct latent subtypes. Second, within the elevated-depressive-symptom profile, symptom network analysis indicated that nodes associated with anger rumination (e.g., ARQ20, ARQ12) and sadness rumination (e.g., SRQ10) showed relatively high expected influence estimates, suggesting their prominent positions in the observed symptom network. Third, descriptive gender-related differences in node centrality were observed.

Overall, these findings extend the understanding of the relationship between emotion-specific rumination and depressive symptoms at both the level of individual classification and symptom interaction, providing preliminary evidence that may inform future intervention development. Furthermore, by differentiating rumination according to emotional content and integrating latent profile analysis with symptom network analysis, this study advances a cross-level analytical framework that links individual heterogeneity with symptom-level interaction mechanisms.

### 4.1 Latent profiles of rumination and depressive symptoms among college students

This study identified three distinct rumination–depressive-symptom profiles, further supporting the notion that rumination and depressive symptoms exhibit pronounced heterogeneity at the individual level (Li et al., 2024; Wytykowska et al., 2022). These results suggest that individuals differ not merely in severity but in the structural configuration of rumination content and emotional experience, rather than conforming to a simple continuous distribution. Among these, the high depressive symptoms–high anger–low sadness rumination subtype was characterized by elevated anger rumination accompanied by pronounced depressive symptoms. This pattern suggests that rumination processes involving different emotional contents may show distinct associations with depressive symptom severity. Anger rumination typically involves repetitive cognitive processing of perceived offenses or injustices. Its core features include strengthened external attributional tendencies (i.e., attributing negative events to others or situational factors) and heightened attentional bias toward hostile information (Takebe et al., 2016). This process not only increases sensitivity to perceived threat cues but also sustains elevated emotional and physiological arousal, thereby maintaining a prolonged state of tension and interpersonal reactivity. In contrast, sadness rumination is more internally oriented, focusing on negative self-referential processing. It reinforces maladaptive self-schemas and feelings of helplessness while simultaneously reducing problem-solving orientation and behavioral activation (Zaid et al., 2025). This inward-focused processing contributes to a persistent low-arousal negative affective state, which is a core feature of depressive symptoms.

Accordingly, anger rumination and sadness rumination may be associated with depressive symptoms through two partially distinct but complementary processes: externally oriented hostile arousal and internally oriented negative self-focus. Evidence further suggests that individuals with elevated depressive symptoms may also exhibit heightened irritability and hostility. In such cases, emotional experience is not limited to low-arousal sadness but is also characterized by increased physiological arousal and outwardly directed negative affect, particularly anger (Voulgaridou, 2025). Under these conditions, anger rumination may become the dominant form of repetitive thinking, whereas sadness rumination plays a relatively reduced role. From an emotion regulation perspective, anger rumination may partially function as a short-term defensive mechanism (Moumne et al., 2021). For individuals experiencing intense self-directed negativity and helplessness (i.e., sadness rumination), shifting cognitive focus from internal failure to external blame may temporarily alleviate self-threat (McLaughlin and Nolen-Hoeksema, 2011). However, this defensive externalization comes at the cost of sustaining emotional arousal and exacerbating interpersonal tension. Over time, such persistent activation may weaken emotional recovery capacity and further aggravate depressive symptomatology through reciprocal reinforcement between cognitive and affective systems.

In summary, the high depressive symptoms–high anger–low sadness rumination subtype may reflect a maladaptive cognitive–emotional pattern characterized by persistent activation and reinforcement of negative repetitive thinking. These findings suggest that depressive symptomatology is influenced not only by the overall level of rumination but also by qualitative differences in rumination content and cognitive processing style.

### 4.2 Network analysis within the elevated-depressive-symptom profile

From the perspective of emotional rumination theory, both ARQ20 (“Whenever I feel angry, I continue thinking about it for a period of time”) and ARQ12 (“I often recall past experiences that made me feel angry”) reflect core features of anger rumination. Specifically, following the induction of negative affect, individuals are unable to disengage from cognitive replay of the triggering event and instead engage in repetitive, passive, and non-constructive processing of anger-related content (Papageorgiou and Wells, 2004). According to the cognitive model of rumination in depression, such repetitive thinking impedes emotional recovery, prolongs negative affective states, and persistently activates maladaptive schemas related to self-defeat and interpersonal hostility, and may be associated with greater depressive symptom severity (Papageorgiou and Wells, 2009). Unlike outwardly expressed anger, anger rumination represents an internally focused form of cognitive processing. Its sustained activation continuously consumes limited cognitive resources, reduces the capacity for adaptive emotional regulation, and may represent a cognitive correlate associated with elevated and persistent depressive symptoms.

In contrast, sadness rumination, as represented by SRQ10 (“I find myself recalling past events that still make me feel sad”), further highlights the role of repetitive negative memory retrieval in depressive symptoms. According to emotion regulation theory, individuals with depressive symptoms tend to repeatedly access negatively valenced autobiographical memories while exhibiting impaired ability to interrupt this process through adaptive strategies such as cognitive reappraisal or attentional disengagement (Burkitt, 2018). This persistent retrieval of negative experiences reinforces negative self-schemas and amplifies feelings of loss and helplessness, thereby trapping individuals in a maladaptive cycle characterized by “negative memory retrieval → reinforcement of negative affect → exacerbation of depressive symptoms.”

Depressive symptoms, sadness rumination, and anger rumination may share variance attributable to repetitive negative thinking. Sadness and anger rumination are also expected to be moderately associated, but they differ in emotional content, attentional focus, and psychological function and therefore need not be redundant. LPA uses multivariate response patterns rather than relying on any single pairwise correlation, whereas the GGM network estimates conditional associations after accounting for the remaining nodes. Shared cognitive variance may still affect profile formation and network estimates, however; future studies should use bifactor models, residual network analysis, or related approaches to separate shared from emotion-specific variance. Therefore, although shared cognitive variance may exist among depressive symptoms, sadness rumination, and anger rumination, it is unlikely to invalidate the interpretation of either the latent profiles or the conditional association network estimated in the present study.

These findings show that anger- and sadness-rumination items were conditionally associated with one another and with aggregate depressive-symptom severity. However, given the cross-sectional design, future longitudinal, experimental, or experience-sampling studies are needed to clarify temporal dynamics and potential causal relationships. In summary, three rumination items had high expected-influence estimates in the present cross-sectional network. These estimates identify potentially informative correlates for future study.

### 4.3 Gender differences in network characteristics within the elevated-depressive-symptom profile

In the male network, SRQ7 (“If something happens that upsets me, I tend to dwell on it”) and SRQ10 (“I recall past events that still upset me”) showed relatively strong expected influence, reflecting pronounced emotional inertia. Specifically, following exposure to negative events, emotional states in males appear less likely to attenuate naturally over time and instead are sustained or intensified through repetitive cognitive replay. Such patterns may be associated with greater depressive symptom severity, although temporal processes require longitudinal investigation. From a dynamic systems perspective, impaired emotional recovery capacity may represent a key marker of increasing vulnerability to depressive symptoms, with rumination representing a potentially important cognitive correlate of depressive symptoms (Eynde and Turner, 2006).

In addition, the prominence of ARQ14 (e.g., “believing that others are worthless”) within the male network may reflect a form of defensive interpersonal hostility. This cognitive tendency may arise from interpersonal frustration or heightened sensitivity to rejection. Such maladaptive appraisals not only distort perceptions of others but may also undermine social connectedness by reducing trust and prosocial motivation (Orchard et al., 2019). Furthermore, this form of defensive hostility may decrease help-seeking behavior and limit the utilization of available social support, thereby increasing the likelihood of social isolation. Given that low social support is a well-established risk factor for both the onset and persistence of depressive symptoms (Paykel, 1994), these findings suggest that sadness-related rumination and interpersonal negative cognitions may represent relevant correlates within the male network.

In contrast, the female network showed a different pattern of relatively high expected-influence estimates. Specifically, ARQ18 (“If something happens that makes me angry, I tend to dwell on it”), ARQ20 (“Whenever I get angry, I keep thinking about it for a period of time”), and SRQ5 (“I keep wondering why I feel sad and find it difficult to think about other things”) all demonstrated high expected influence, indicating a dominant pattern of attentional fixation. In this process, attentional resources are persistently consumed by negative emotional content, thereby limiting the capacity for attentional disengagement. This sustained attentional bias promotes the dominance of negative information within the cognitive system while weakening the processing of positive stimuli, which may be associated with sustained negative cognitive processing. Over time, such bias not only exacerbates emotional distress but may be related to greater depressive symptom severity.

Notably, the central role of anger rumination (e.g., ARQ18 and ARQ20) in the female network suggests that anger rumination is not merely a passive form of recollection but may also involve the amplification and reactivation of emotional experiences. This “emotional amplification effect” may intensify initially moderate negative emotions, leading to sustained psychological strain, internal tension, and cumulative emotional burden, thereby increasing vulnerability to depressive symptoms. Meanwhile, SRQ5 reflects the excessive occupation of cognitive resources by emotional processing, which may be associated with difficulties in adaptive cognitive regulation. According to motivational and self-regulation theories, prolonged goal disruption can generate feelings of helplessness and diminished perceived control, both of which are key psychological mechanisms implicated in the development and persistence-related symptom associations of depressive symptoms (Vancouver, 2008).

### 4.4 Implications for practice

The findings provide several implications for prevention-oriented mental health practice among college students.

First, identifying emotion-specific patterns of rumination may enhance the understanding of individual differences in depressive symptom experiences. Considering both anger-related and sadness-related rumination in psychological assessment may provide additional information for identifying students who exhibit different cognitive-emotional patterns associated with elevated depressive symptoms.

Second, the rumination items showing relatively high expected-influence estimates may represent important cognitive-emotional features that warrant further attention in preventive programs. Interventions focusing on adaptive emotion regulation, reducing repetitive negative thinking, and promoting more flexible cognitive processing may help address maladaptive rumination patterns associated with depressive symptoms.

Third, the identification of heterogeneous rumination–depressive-symptom profiles highlights the importance of personalized approaches in college mental health prevention. Rather than applying a uniform intervention strategy, prevention programs may benefit from considering individual differences in rumination content and depressive symptom patterns when designing psychological education and support services.

### 4.5 Limitations and directions for future research

Several limitations of this study should be acknowledged.

First, the sample was recruited from a single university in a specific geographic region, which may limit the generalizability of the findings. Although the relatively large sample size provided adequate statistical power, future studies should replicate these findings across multiple regions, educational contexts, and more diverse populations to enhance external validity.

Second, the sample contained a higher proportion of female participants than male participants (69.4% vs. 30.6%). Although gender was not included as an indicator in the latent profile analysis and the profiles were estimated in the pooled sample, this imbalance may have influenced the relative prevalence and estimation precision of the identified profiles. In addition, unequal gender group sizes may have affected the statistical power and comparability of gender-stratified network analyses. Therefore, gender-related findings should be interpreted cautiously as exploratory patterns rather than definitive gender-specific mechanisms. Future studies should recruit more gender-balanced samples and further examine these patterns using multi-group latent profile analysis and network approaches.

Third, although the present network analysis identified important symptom associations, additional network methods, such as bridge symptom analysis and community detection, may provide further insight into the organization of depressive symptoms and rumination dimensions. Moreover, because the present study was cross-sectional, longitudinal and experience-sampling designs are needed to clarify temporal dynamics and potential causal relationships among rumination and depressive symptoms.

## Data Availability

The raw data supporting the conclusions of this article will be made available by the authors, without undue reservation.
