# Supplementary material for: A study on the symptomatic association between depressive symptoms and rumination in college students–based on latent profile analysis and network analysis
Source: Front Psychol. 2026 Aug 12;17:1875520. doi: 10.3389/fpsyg.2026.1875520 (PMC13506428; doi:10.3389/fpsyg.2026.1875520)
Supplement: Supplementary file 1 [file Table_1.docx]

**Supplementary Figure S1：**

**Edge-weight accuracy**

**Supplementary Figure S2：**

**Centrality-difference test**

**Supplementary Figure S3：**

**Case-dropping stability**

**Supplementary Table S1：**

**Complete edge-weight matrix**

**Supplementary Table S2：**

**Edge weights and bootstrap confidence intervals**

**Supplementary Table S3：**

**Centrality-difference results**
